# Interactions between Radiation and One-Carbon Metabolism

**DOI:** 10.3390/ijms23031919

**Published:** 2022-02-08

**Authors:** Navyateja Korimerla, Daniel R. Wahl

**Affiliations:** 1 Department of Radiation Oncology, University of Michigan, Ann Arbor, MI 48109, USA; korimer@med.umich.edu; 2 Rogel Cancer Center, University of Michigan, Ann Arbor, MI 48109, USA

**Keywords:** one-carbon metabolism, radiation therapy, cancer therapy, folate cycle, methionine cycle

## Abstract

Metabolic reprogramming is a hallmark of cancer. Cancer cells rewire one-carbon metabolism, a central metabolic pathway, to turn nutritional inputs into essential biomolecules required for cancer cell growth and maintenance. Radiation therapy, a common cancer therapy, also interacts and alters one-carbon metabolism. This review discusses the interactions between radiation therapy, one-carbon metabolism and its component metabolic pathways.

## 1. Introduction

Radiation therapy (RT) is a frequently used cancer therapy that kills cells by inducing oxidative damage to macromolecules including DNA [1]. Altered metabolism is a hallmark of cancer [2]. Many cancers exhibit altered metabolic reprogramming of one-carbon metabolism, which is a series of interlinked pathways that can convert dietary inputs such as folic acid and methionine into useful biochemical outputs including nucleotides, antioxidants and methyl units that serve as cornerstones of cellular homeostasis [3]. Exposure to radiation disrupts normal cellular homeostasis thus altering cellular metabolism [4]. While damaged DNA appears to be the most critical mediator of RT-induced cell death, radiation-induced metabolic changes are particularly noteworthy as metabolism is closely related to cellular phenotype and they undergo robust changes in response to external perturbations such as radiation [5,6,7]. Further, scientific advancement in tools such as nuclear magnetic resonance (NMR) spectroscopy and mass spectrometry (MS) has enabled the study of how radiation and metabolism are linked [4,5]. Here, we discuss the links between RT and the various nodes of one-carbon metabolism. In particular, we highlight how RT affects the activity of one-carbon metabolic pathways and how the activity of these pathways regulates the radiation response. Because of the importance of one-carbon metabolism for the growth and survival of cancers, understanding the links between RT and one-carbon pathways could help develop new cancer-specific combination therapeutic strategies.

## 2. One-Carbon Metabolism: A Short Overview

**Figure 1:**
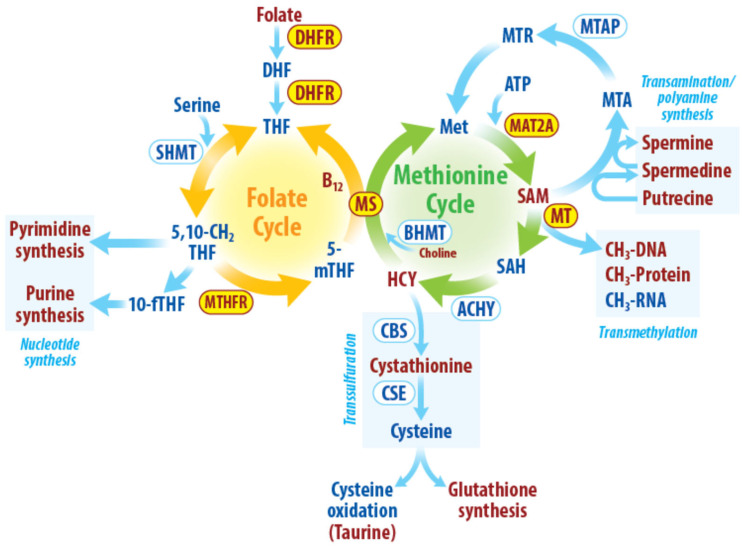
Overview of radiation-induced changes in one-carbon metabolism. Enzymes, metabolites and pathways affected by radiation directly or indirectly are highlighted in the figure. Dihydrofolate (DHF), Tetrahydrofolate (THF), serine hydroxymethyltransferase (SHMT), 10-formyltetrahydrofolate (10-fTHF), 5-methyltetrahydrofolate (5-mTHF) methylenetetrahydrofolate reductase (MTHFR), methionine synthase (MS), methionine adenosyltransferase 2A (MAT2A), S-adenosylmethionine (SAM), methyltransferases(MT), S-adenosylhomocysteine (SAH), adenosylhomocysteinase (ACHY), methylthioadenosine phosphorylase (MTAP), 5′-Methylthioadenosine (MTA), 5-deoxy-5-(methylthio)ribose (MTR), betaine-homocysteine methyltransferase (BHMT), homocysteine (HCY), cystathionine β-synthase (CBS), cystathionine γ-lyase (CSE). Enzymes, metabolites and pathways affected by radiation directly/indirectly are highlighted in red.

One-carbon metabolism encompasses a series of interlinked metabolic pathways [3,8]. One-carbon metabolism has various nutritional inputs including glucose, serine, threonine, methionine, glycine, choline, folate, and vitamin B12. An overview of one-carbon metabolism and its constituent metabolic pathways is shown in Figure 1. One-carbon units from a major methyl donor, dietary folate, are transferred via a series of reactions to various methyl acceptors and is also recycled via by-product homocysteine by two cycles termed the methionine and folate cycles.

In the methionine cycle, the amino acid methionine combines with ATP to generate S-adenosyl methionine (SAM) in a reaction catalyzed by methionine adenosyltransferase 2A (MAT2A). After donating its methyl group to a substrate, SAM, the universal methyl donor, is converted to S-adenosylhomocysteine (SAH). SAH is then hydrolyzed by adenosylhomocysteinase to make homocysteine. Homocysteine can be recycled to methionine by cobalamin-dependent methionine synthase (MS). The MS reaction utilizes a folate-cycle derived methyl group on 5-methyltetrahydrofolate (5 m THF) to regenerate methionine, thus connecting the methionine and folate cycles [9].

In the folate cycle, folate is converted to tetrahydrofolate (THF) by dihydrofolate reductase (DHFR). THF is converted to 5,10-methylene THF by serine hydroxymethyltransferase-2 in the presence of serine. 5,10-methylene THF directly contributes to pyrimidine synthesis. Its conversion into 10-formyltetrahydrofolate (10-fTHF) by methylenetetrahydrofolate dehydrogenase 1 (MTHFD1) allows it to contribute methyl groups to the de novo synthesis of purines. Alternatively, methylenetetrahydrofolate reductase (MTHFR) can convert 5,10-methylene THF into 5-methyltetrahydrofolate (5-mTHF), which serves as a one-carbon donor to regenerate methionine from homocysteine and simultaneously yields THF in the folate cycle.

The metabolic pathways driven by one-carbon metabolism include transmethylation, transsulfuration, transamination, and nucleotide synthesis. These metabolic pathways are essential for cellular functions such as epigenetic regulation, redox maintenance, and biomolecule synthesis. Transmethylation involves the transfer of methyl groups from SAM, a ubiquitous methyl donor, to a variety of biomolecules such as DNA, RNA, proteins, and lipids. These transmethylation reactions are critical for numerous aspects of biology including the maintenance and regulation of the epigenome [10,11]. In the transsulfuration pathway, homocysteine from the methionine cycle provides the sulfur for cystathionine formation. Cystathionine is then converted to cysteine and subsequently, glutathione, a major cellular antioxidant required for redox homeostasis [12]. Transamination provides a critical step in polyamine synthesis. SAM acts as a donor of aminopropyl groups in polyamine synthesis [13]. One-carbon metabolism is also required for nucleotide synthesis as methylated forms of folate are essential during the de novo synthesis of both pyrimidines and purines [3]. 

In summary, one-carbon metabolism converts dietary inputs into useful cellular building blocks and is crucial for a variety of cellular processes such as growth, development, and differentiation [14]. Aberrant one-carbon metabolism can lead to pathological conditions such as cancer, cardiovascular and neurological diseases [3]. Many of the reactions of one-carbon metabolism are influenced by radiation therapy (RT). These interactions are summarized in Table 1 and described in detail below.

## 3. Interactions between Radiation and the Folate Cycle

Folate is an essential nutrient obtained only through the diet. RT damages cellular biomolecules and folate is no exception. Folate is particularly susceptible to RT and is easily degraded through radiolysis after radiation exposure. Folic acid is composed of a pteridine ring, p-aminobenzoic acid, and glutamate. While high doses of RT (up to 10 KGy using electron beams) degrade the majority of folate in isolated aqueous solutions, lower doses that are more relevant to therapeutic radiation (i.e., 3 Gy) can also cause meaningful folate degradation [25]. These changes are also seen in more complex biological systems. In mice, total body irradiation of 3Gy causes a drastic drop of plasma and bone marrow folate levels that begins several hours after RT and lasts for several days [15]. Consistent with direct RT-induced degradation of folate (as opposed to increased consumption), total body irradiation of mice also increases the levels of the folate degradation product P-amino benzoyl glutamic acid [26]. Observational studies in cancer patients undergoing therapeutic RT suggest that similar RT-induced folate degradation occurs in humans [16]. However, the patients in this study did not show clinical symptoms of folic acid deficiency suggesting radiolysis of folic acid might be less important than other effects of RT on the folate cycle.

In addition to direct effects on the folate molecule, RT can alter the activity of the folate cycle. Whole-body irradiation in folate-depleted mice reprograms one-carbon flux and reduces SAM reserves [22]. Exposure to radiation in mice increases the activity of DHFR and thymidylate synthase (TS) in the liver in the days after radiation, while MTHFR activity is reduced [17]. These changes suggest that, following RT, tissues prioritize folate-dependent thymidine synthesis at the expense of folate-dependent transmethylation. However, this RT-induced reduction in methylation is not a universal phenomenon and seems to only happen when RT is given as total body irradiation (TBI) which limits folate, leading to diversion of folate pools towards nucleotide synthesis at the expense of transmethylation. In other contexts, RT can activate methylation reactions, and this is discussed in detail later in this text (interactions between radiation and transmethylation reactions).

While RT modulates folate metabolism, folate metabolism also regulates the effects of RT. The activity of the folate cycle is important for preventing and repairing DNA damage by altering nucleotide metabolism. The folate cycle provides the building blocks for the synthesis of purines and pyrimidines. 5,10-methylene THF is required for de novo pyrimidine synthesis and 10-fTHF from the folate cycle is required for de novo purine synthesis. Thus, the folate cycle plays a key role in DNA and RNA synthesis in a cell [27]. Thymidylate synthase (TS), which catalyzes the conversion of deoxyuridine monophosphate to deoxythymidine monophosphate, is a rate-limiting enzyme in DNA synthesis. Depletion of folate in cells results in increased, dispersed but not clustered gH2AX foci (indicative of DNA damage). Folate deficiency not only induces DNA damage but also shifts DNA to euchromatin state which gives access to DNA damage repair factors [28]. Folate replenishment rescued this phenotype by forming clustered and dispersed gH2AX foci and recovering the DNA to heterochromatin state, suggesting repair of DNA damage. In Chinese hamster ovary cells, folate deficiency by itself can cause DNA double-stranded breaks, and further, folate deficiency in combination with radiation both increases radiation-induced DNA damage and slow its repair [29]. Similarly, in rats, folate deficiency causes DNA double-stranded breaks and slows DNA repair by interfering with p53 [30]. Consistent with these effects on the induction and resolution of RT-induced DNA damage, folate deficiency also increases radiation-induced micronuclei formation [30].

The mechanism through which folate deficiency inhibits DNA repair is by uracil misincorporation [31]. Folate deficiency results in an imbalance in the nucleotide precursor pool and thereby leading to misincorporation of uracil in the newly synthesized DNA generated to repair RT-induced DNA damage [32]. Uracil misincorporation leads to DNA double-stranded breaks and chromosomal damage [32]. In addition to altering thymine/uracil balance, folate metabolism regulates purine pools. Because purines promote DNA repair in a variety of systems including brain tumors, it is possible that folate metabolism regulates DNA repair through purine in addition to pyrimidine metabolism [33]. Folate supplementation, on the other hand, reduces ionizing radiation-induced DNA damage and genomic instability thereby decreasing the fraction of apoptotic cells indicating a radioprotective effect of folate supplementation in vitro [34]. Whether folate supplementation promotes DNA repair through thymine/uracil metabolism or other mechanisms is not known.

The interactions between RT and the folate cycle can be exploited clinically. Methotrexate (MTX), a widely utilized anticancer agent, is a competitive inhibitor of DHFR [35]. MTX also inhibits other enzymes in nucleotide synthesis including thymidylate synthase and GART, an enzyme used to synthesize purines [36]. MTX radiosensitizes a variety of cancers but is rarely combined with RT in patients due to high risks of toxicities [37,38]. For example, combining high dose methotrexate with radiation is effective for patients with primary CNS lymphoma, but can cause severe delayed neurotoxicity [39]. Similarly, combined radiation and methotrexate can increase the risk of neurologic toxicity in patients with acute lymphoblastic leukemia [40] and skin toxicity in patients with head and neck cancer [41]. In patients with hematologic malignancies methotrexate with radiation can increase the risk of myelopathy [42]. Pemetrexed disodium, which inhibits several folate-dependent enzymes in purine and pyrimidine synthesis, is also a radiosensitizer in laboratory and clinical settings [43,44]. In summary, folate metabolism, especially the folate-dependent synthesis of thymidine, is intimately related to the radiation response and its modulation can influence RT responsiveness.

## 4. Radiation at the Interface of the Folate and Methionine Cycles

The folate cycle is linked to the methionine cycle and their interaction is essential for the recycling of homocysteine to methionine. Cobalamin (Vitamin B12), an essential vitamin, and betaine, a modified amino acid, serve as cofactors in the conversion of homocysteine to methionine. RT depletes total body cobalamin, likely by reducing intestinal absorption of B12. After total body irradiation of normal and tumor-bearing mice, plasma B12 levels dropped in both groups, while homocysteine levels increased only in those bearing tumors, indicating that RT reduces cobalamin levels regardless of the presence of cancer [18]. In patients with rectal cancer, cobalamin levels in the serum decrease rapidly after pelvic radiotherapy [19]. Pelvic or abdominal radiotherapy given to patients with bladder, rectal and gynecological cancer may cause damage to the small intestine resulting in reduced absorption of cobalamin and subsequent discoordination of the folate and methionine cycles.

Betaine, derived from choline, is an important factor for the conversion of homocysteine to methionine by BHMT (betaine-homocysteine methyltransferase). In other words, betaine acts as a methyl donor used to generate methionine independently of the folate cycle. Tumor and normal cells seem to have differential expression of BHMT, and hence have differential flux towards methionine regeneration [45]. This alternative source of carbons for methionine regeneration becomes increasingly important in irradiated tissues to allow cells to simultaneously fuel both the folate and methionine cycles. While RT can increase cellular choline demands to repair damaged phosphatidylcholine molecules in cell membranes, this does not deplete total choline reserves in choline-sufficient mice [20]. However, in mice depleted of either choline or folate, clinically relevant doses of TBI cause the release of choline reserves from the liver, in part to fuel choline metabolism in the brain [20,21]. Thus, RT appears to trigger the release of hepatic choline reserves to be utilized by other organs for RT-protection when nutrition is limited [21]. Consistent with the hypothesis that choline and betaine protect cells from RT, betaine supplementation protects lymphocytes from chromosomal aberrations in vitro and increased the lifespan of mice by protecting the bone marrow and intestines from radiation damage [46,47]. In summary, total body irradiation in mice seems to mobilize hepatic choline reserves under choline or folate deprivation while choline supplementation provides radioprotection. By serving as a folate-independent source of one-carbon units, betaine and choline allow tissues to engage in both the folate-dependent and methionine-dependent reactions needed to detoxify RT-induced cellular damage.

## 5. Interactions between Radiation and the Methionine Cycle

Methionine is an essential amino acid and, like folate, must be obtained from the diet. Numerous tumors are dependent on methionine metabolism for growth and survival, a phenomenon termed the Hoffman effect, and the activity of methionine metabolism can provide both diagnostic and prognostic information in cancer [48,49,50,51,52,53]. For example, ^11^C-methionine is used for positron emission tomography to differentiate recurrent brain tumors from necrotic tissue [53]. A clinical trial is undergoing to test if [^11^C]-L-Methionine can be used to help diagnose central nervous system (CNS) tumors and sarcomas (NCT00840047). Metabolically active cancer cells have an increased demand for transmethylation reactions and hence have enhanced methionine dependency [54,55].

Methionine dependency in cancers has several causes. Many cancers harbor a homozygous deletion of the gene encoding methylthioadenosine phosphorylase (*MTAP*), a key enzyme in the methionine salvage pathway. This deletion occurs due to the proximity of *MTAP* to the tumor suppressor *CDKN2A* and its loss prevents the conversion of methylthioadenosine to methionine [56]. Methionine dependency in cancer can also be caused by low levels of methionine synthase, the enzyme that catalyzes the conversion of homocysteine to methionine [57,58]. Finally, PI3KCA mutations lead to methionine dependency by decreasing expression of the cysteine transporter, thereby diverting homocysteine into the transsulfuration pathway rather than using it to regenerate methionine [59,60].

How radiation interacts with the methionine cycle is incompletely understood. Radiation induces systemic changes in methionine uptake by different organs. Exposure to RT as low as 3Gy leads to a significant and persistent reduction in methionine in mouse jejunum [61]. The reduction in methionine levels post-irradiation causes DNA hypomethylation and also impairs glutathione synthesis needed to counteract RT-induced oxidative damage. Consistent with these findings, in a study comparing the uptake of radiolabeled methionine in different tissues 6 days post total body irradiation (7Gy), uptake of methionine-2-^14^C and methionine methyl-^14^C was less in the brain, heart, muscle, and kidney in irradiated subjects compared to the controls [62]. Interestingly, while radiolabeled protein from methionine-2-^14^C was lower in these tissues, ^14^C from methionine methyl-^14^C was higher in tissue proteins compared to the control subjects suggesting that the radiation alters the consumption of the methyl group of methionine by these tissues. In addition, radiolabeled methionine was given six days post-irradiation suggesting the observed changes are long-term changes upon radiation-induced tissue injury and not acute, adaptive RT-induced changes. Consistent with these findings, proton and ^56^Fe RT (0.1Gy-0.5Gy) modestly reduces methionine-derived SAM levels at 6 days, but once tissue recovers, SAM levels recover as well. In summary, RT reduces methionine levels as tissue is damaged, but these levels recover upon tissue recovery. The acute impact of RT on methionine metabolism is not clearly understood.

Dietary intervention can overcome genetic influences and markedly change cellular metabolism [63]. Methionine supplementation increases both MAT2A and MS activity increasing cellular SAM levels [23]. Methionine supplementation also increases methyltransferase activity and helps maintain DNA methylation following irradiation [23]. While methionine supplementation appears to reduce the negative effects of radiation, contradicting evidence shows that methionine diet supplementation worsens radiation-induced gastrointestinal syndrome likely due to bacterial overgrowth and impairments in gut physiology [64]. In total body irradiated mice fed with a normal diet, radiation reduces hepatic folate and choline, thereby reducing SAM levels [24]. However, DNA methyltransferase activity and methionine synthase activity remain unchanged. Methionine supplemented diet enhances methionine synthase activity and DNA methyltransferase activity causing DNA hypermethylation 24 and 48 h post-irradiation [24]. This study indicates hepatic folate and choline mobilization after total body irradiation into the serum. However, it is not clearly understood whether this phenomenon is tissue-specific or a universal tissue injury response to radiation. Further studies evaluating the effect of radiation on acute changes in the methionine cycle in cancers and normal tissues are needed to fully understand these changes.

Dietary methionine restriction, like methionine supplementation, can also alter methionine and sulfur metabolism. Dietary methionine restriction appears to improve radiation efficacy [61]. Methionine-related metabolites reduce within two days of methionine restriction and sustain throughout diet restriction. Methionine diet restriction both inhibits tumor growth and radiosensitizes RAS-driven sarcoma models [65,66]. Methionine restriction also improves the efficacy of chemotherapeutic drugs. For example, in tumor-bearing rats, depleting methionine increased the efficacy of vincristine [67]. In gastric cancer patients, there is preliminary data that methionine-free amino acid supplementation potentiates the effects of 5-Fluorouracil [68]. An alternative strategy to reduce methionine levels is by using methioninase, an enzyme that rapidly depletes methionine levels. Methioninase enhanced the efficacy of chemotherapy in mice bearing human colon cancer, Ewing’s sarcoma, melanoma, and brain tumors [69,70,71,72]. Since chemotherapy and radiotherapy are both genotoxic agents, there remains an underexplored opportunity to use methionine diet restriction to potentiate radiotherapy. Even though several preclinical studies show that dietary methionine restriction potentiated the effects of genotoxic agents, few clinical trials exploit methionine diet restriction with chemotherapy or radiotherapy. A phase II clinical trial with methionine diet restriction showed increased efficacy of chemotherapy in melanoma and recurrent gliomas [73]. A phase I clinical trial to exploit methionine diet restriction with radiation in cancers is currently ongoing (NCT03574194). Should results from these studies show promise, randomized studies of the methionine-restricted diet will be needed to formally assess its efficacy.

## 6. Radiation and Transmethylation

One-carbon metabolism provides the methyl carbons required for transmethylation reactions. SAM is a universal methyl donor and is a substrate for a variety of methyltransferases. MAT2A catalyzes the conversion of methionine to SAM. SAM acts as a substrate to methyltransferases and upon transfer of methyl group is converted to SAH. SAH inhibits SAM-dependent methyltransferases. The SAM/SAH ratio is an indicator of cellular methylation potential. The cellular methylation potential dictates transmethylation reactions that are essential for DNA, histone and protein methylation. Hence, one-carbon metabolism profoundly influences the post-translational and epigenetic regulation of the cellular state [10,74]. Like the other pathways in one-carbon metabolism, transmethylation reactions are regulated by RT and help modulate the cellular response to RT.

RT causes specific methylation changes in global DNA, specific genes and repetitive genomic elements [75]. DNA methylation exclusively occurs on the cytosine residues located in the cytosine and guanine base sequences (CpG). The majority of these methylated sites are located in either short (<4 Kb) regions of DNA (that contain large numbers of CpGs) or in long domains of predominantly repetitive DNA elements. RT (0.5–10 Gy) induced global DNA hypomethylation 24, 48 and 72 h after exposure in cell culture models [76]. However, animal studies showed that the RT-induced DNA hypomethylation changes are not universal and are tissue and sex specific [77]. For example, whole body irradiation decreases DNA methylation in mouse liver within hours. However, similar hypomethylation was not observed in the DNA of brain, spleen and cultured cells [78]. In a study comparing DNA methylation pattern in radiation sensitive and resistant cell lines, RT caused differential DNA methylation in a time dependent fashion [79]. In general, RT induces global DNA hypomethylation and hypermethylation of tumor suppressor genes as discussed in detail elsewhere [75]. RT-induced DNA hypomethylation may also mediate genomic instability and carcinogenesis [80]. For example, RT reduces the DNA methylation 5′-UTRs of LINE-1 retrotransposons (which regulate gene expression and drive evolution) thereby leading to aberrant expression of these mediators of genetic instability [81]. In summary, RT-induced DNA methylation seems to vary by time, genetic background, tissue of origin, and sex. RT also induces transcriptional modifications, post-transcriptional modifications, and post-translational modifications [82].

In addition to regulating the methylation of DNA itself, RT alters the methylation of histones, thereby providing another layer of biologic regulation [83]. For example, RT reduces methylation of H4-Lys20 which is accompanied by persistence of histone γH2AX foci indicative of DNA damage [84]. The relaxed chromatin state induced by H4-Lys20 hypomethylation is required for recruitment of DNA repair factors. On the other hand, methylation of histones (H3K9) after DNA damage is required for homology directed repair, by promoting recruitment of Tip60 and ATM to the site of DNA damage [85]. Similarly, methylation of histone (H3Lys36) near the double stranded breaks results in accumulation of Ku70-associated DNA-PK required for efficient DNA damage repair [86]. More and more evidence indicates the critical role of histone methylation for efficient DNA damage repair [87]. Together, these findings suggest that both histone methylation and demethylation are needed for efficient DNA repair after RT and suggest that interfering with these reactions may potentiate the efficacy of RT.

Modulation of one carbon metabolism can alter cellular transmethylation thereby influencing RT efficacy. Altering methionine levels by diet restriction can alter the metabolic flux through the methionine cycle and modulate the chromatin state in cells [88]. Specifically, methionine metabolism can directly alter SAM and SAH levels, robustly changing the methylation on histones (H3K4) [88]. Methionine availability not only alters global H3K4 methylation but also influences gene expression by altering H3K4 peak width [89]. Modulation of SAM levels directly by methionine restriction or indirectly by reducing SAM synthesis can both alter the flux through methionine cycle towards transmethylation reactions [89,90]. Similarly, pharmacological inhibition of MAT2A not only reduces cellular SAM levels but also reduces transmethylation by a methyl transferase, PRMT5 on arginine residues [91]. On the other hand, methionine supplementation alters RT-induced one-carbon metabolic flux increasing hepatic SAM, resulting in a higher and persistent methylation potential [23]. In summary, regulation of methionine levels can alter the chromatin state in cells by interfering with SAM and trimethylation. RT can also differentially regulate MAT2A and MS activity upon methionine supplementation and account for increased flux through one-carbon metabolism [23,24]. RT-induced transmethylation reactions alter DNA and protein methylation and theses transmethylation reactions may in turn dictate RT efficacy. Methylation of histones at the site of DNA damage not only helps in recruitment of DNA repair factors required for DNA damage repair, but also dictate the choice of DNA damage repair pathway and are extensively reviewed elsewhere [87]. A recent study showed that reduction in SAM levels using a pharmacological inhibitor of MAT2A reduces dimethylation on arginine residues causing a splicing aberration in DNA repair factors that are crucial for efficient DNA damage repair [91]. RT-induced acute methylation changes that may be required for efficient DNA damage repair are not well studied. Because of the critical role that trimethylation plays in the RT response, additional work is needed to determine how interfering with transmethylation affects the efficacy of RT and the DNA damage response.

## 7. Radiation and Polyamine Synthesis

Polyamines (including putrescine, spermidine, and spermine) are aliphatic cations and interact with biomolecules both electrostatically and covalently. Polyamines play a critical role in the normal growth and function of cells by regulating a variety of cellular processes such as proliferation, metabolism, and differentiation [13]. A critical step in polyamine synthesis is linked to one-carbon metabolism through transamination. In the methionine salvage pathway, SAM is converted to decarboxylated S-adenosylmethionine (dcSAM) by adenosylmethionine decarboxylase. dcSAM acts as a donor of aminopropyl groups and aids in polyamine synthesis. The aminopropyl groups from dcSAM can be used to convert putrescine to spermidine by spermidine synthase and to convert spermidine to spermine by spermine synthase.

Polyamines undergo robust changes in response to RT. TK6 cells exposed to RT doses of 0.5 to 8.0 Gy showed time and dose-dependent reductions in spermine levels as early as 1hr post-RT. These metabolic changes are consistent in a transformed fibroblast cell line, BJ [92]. Similar findings are seen in rats, especially in highly proliferating tissues such as the spleen and small intestine, which decrease polyamine levels following TBI [93]. This reduction suggests a block of cell proliferation post-irradiation as polyamines are essential for cell growth [94].

Polyamines detected in urine and red blood cells of RT-treated cancer patients were significantly higher than normal healthy controls. Polyamines in pelvic irradiated prostate cancer patients showed a temporal profile with low polyamine levels related to favorable outcomes. Hence, polyamines may be indicators of radiation injury [95]. Lastly, the polyamine levels increased during the late phase of tissue injury and returned to normal levels after tissue recovery from radiation [93,95,96]. In summary, polyamines reduced acutely after RT and increased during the recovery phase.

RT-induced alterations in polyamine metabolism promote DNA damage repair and blunt RT-induced oxidative damage. In a metabolic screening performed with young and old irradiated mice, younger mice were better able to elevate polyamines following RT compared to their older counterparts. This impaired elevation of polyamines in older mice results in reduced DNA repair following RT [97]. Polyamines aid in RT-induced DNA damage repair by promoting homology-directed repair without affecting non-homologous end joining by promoting DNA strand exchange by Rad51 [98]. Knockdown of a key polyamine catabolic enzyme, SAT1, sensitized GBM cells to radiation and reduced tumorigenesis in vivo [99]. Low levels of polyamines in cells increased the sensitivity to oxidative damage [100]. RT generates reactive oxygen species and induces oxidative damage to a cell. Oxidative damage also increases the uptake of polyamines [101]. Overall, both the antioxidant and DNA repair-stimulating properties of polyamines appear important for their ability to protect against RT-mediated cellular damage [102,103].

## 8. Radiation and Transsulfuration

Elevated levels of homocysteine are associated with increased cancer aggressiveness. For example, compared to lower grade tumors, high-grade renal cell carcinomas have elevated methionine cycle products, including SAM, SAH and homocysteine [92]. RT can further elevate the homocysteine levels in cancers. In tumor bearing mice, RT increases plasma homocysteine levels [18]. RT-induced increased homocysteine is diverted towards the transsulfuration pathway, to produce more cystathionine, without changing metabolites in homocysteine remethylation [93]. Further, RT can also increase transsulfuration-derived cellular GSSG levels. Plasma GSSG levels elevate as early as 10 min following RT and persist for hours in irradiated mice [104]. Similarly, blood GSSG is also elevated in breast and lung cancer patients receiving radiotherapy [104]. Together, these results indicate robust GSH consumption and GSSG production following RT, which would require increased activity of the methionine cycle and transsulfuration pathways to replenish GSH pools, especially when alternative sources of cysteine are lacking [105,106].

The transsulfuration pathway is essential for cancer cell growth under nutrient limitation [107]. Impairment of transsulfuration activity can potentiate the effects of RT. Methionine diet restriction reduces homocysteine and cystathionine levels and sensitizes Ras-driven autochthonous sarcomas to RT, possibly through alteration in the redox balance mediated by one-carbon metabolism [65]. Direct inhibition of the transsulfuration pathway by inhibition of cystathionine-β-synthase (CBS) or cystathionine-γ-lyase (CES) might prove useful therapeutic targets to radiosensitize cancers that are transsulfuration-dependent. Cellular transmethylation is recently described as the rate limiting steps of transsulfuration pathway [107]. Strategies indirectly targeting transsulfuration by acting on this rate limiting step, either by nutrient deprivation or by using pharmacological inhibitors, might prove useful to improve RT efficacy.

## 9. Conclusions

Metabolic reprogramming is a hallmark of cancer. One-carbon metabolism is a central metabolic hub that is essential for nucleotide synthesis, polyamine synthesis, epigenetic maintenance and redox balance. Tumor-specific one-carbon metabolism can be exploited to selectively target tumor-specific vulnerabilities and is an attractive target in cancer therapy [108]. RT interacts with one-carbon metabolism at multiple nodes and induces changes in one-carbon metabolism. Radiation-induced changes in the folate cycle and methionine cycle of one-carbon metabolism are highly interconnected and complex. While extensive preclinical work answers basic questions of radiation-induced changes in one-carbon metabolism, the majority of preclinical studies use total body irradiation to study the effects of radiation on one-carbon metabolism. Total body irradiation can give a general understanding of tissue damage response and changes in the one-carbon network. However, radiation is typically administered focally in the clinic to maximize anti-cancer efficacy while minimizing normal tissue toxicity. Radiation-induced one-carbon metabolic changes could be different in clinical settings where radiation is given as a focal therapy to specifically target the tumor cells and spare healthy cells from the toxic effects of radiation. Further studies are needed to understand tumor-specific one-carbon metabolic changes upon focal radiotherapy. Understanding radiation-induced changes in one-carbon metabolism is critical for the exploitation of one-carbon metabolic inhibitors to overcome RT resistance. Inhibition of the folate cycle along with RT have long been used in clinical settings to improve RT effectiveness [109]. New therapeutic strategies to intervene upon methionine metabolism through dietary or pharmacologic hold promise to augment the efficacy of RT. Our growing mechanistic understanding of how the RT response and one-carbon metabolism are linked should facilitate the clinical development of combination therapies using RT and modulation of these pathways.

## Figures and Tables

**Table 1:** Overview of effects of radiation on one carbon-metabolism.

One-Carbon Pathway	Radiation	Dose	Effects	Model	Reference
Folate cycle	Total body irradiation	3 Gy	Reduces folate levels	Mice	[15]
	Therapeutic RT		Reduces folate levels	Cancer patients	[16]
	Total body irradiation	2–7 Gy	Increases DHFR activity	Mice	[17]
	Total body irradiation	2–7 Gy	Increases Thymidylate synthase (TS) activity	Mice	[17]
	Total body irradiation	2–7 Gy	Reduces MTHFR activity	Mice	[17]
Interface of methionine and folate cycle	Total body irradiation	5.6–9.6 Gy	Reduces cobalamin levels	Mice	[18]
	Pelvic radiotherapy	50 Gy	Reduces cobalamin levels	Rectal cancer patients	[19]
	Total body irradiation	2–6 Gy	Reduces hepatic choline levels	Choline-free diet (CFD) mice	[20]
	Total body irradiation	1–4 Gy	Mobilizes hepatic choline reserves to other organs	folate free diet (FFD) mice	[21]
Methionine cycle	Total body irradiation	2–4 Gy	Depletion of SAM levels	folate free diet (FFD)mice	[22]
	Total body irradiation	2–4 Gy	Altered MAT2A activity	L-methionine supplemented diet (MSD) mice	[23]
	Total body irradiation	2–4 Gy	Altered MS activity	L-methionine supplemented diet (MSD) mice	[23]
	Total body irradiation	2–6 Gy	Increased Methyl transferase activity	methyl-supplemented diet (MSD) mice	[24]

## Data Availability

Not applicable.
